# Porcine Sample Type Characteristics Associated with Sequencing and Isolation of Influenza A Virus

**DOI:** 10.3390/vetsci12070683

**Published:** 2025-07-19

**Authors:** Daniel C. A. Moraes, Onyekachukwu H. Osemeke, Michael A. Zeller, Amy L. Baker, Gustavo S. Silva, Giovani Trevisan, Daniel C. L. Linhares, Phillip C. Gauger

**Affiliations:** 1 Department of Veterinary Diagnostic and Production Animal Medicine, College of Veterinary Medicine, Iowa State University, Ames, IA 50011, USA; moraes@iastate.edu (D.C.A.M.); oosemeke@iastate.edu (O.H.O.); mazeller@iastate.edu (M.A.Z.); gustavos@iastate.edu (G.S.S.); trevisan@iastate.edu (G.T.); linhares@iastate.edu (D.C.L.L.); 2 Virus and Prion Research Unit, National Animal Disease Center, USDA-ARS, Ames, IA 50010, USA; amy.l.baker@usda.gov

**Keywords:** influenza A virus, Sanger sequencing, virus isolation, swine, diagnostics

## Abstract

Understanding how sample type may influence the probability of influenza A virus (IAV) sequencing and isolation success can help improve the use of diagnostic tests and refine surveillance strategies in swine populations. The objective of this study was to evaluate the probability of success for IAV hemagglutinin (HA) and neuraminidase (NA) Sanger sequencing and virus isolation in Madin–Darby Canine Kidney (MDCK) cells across different porcine sample types submitted to the Iowa State University Veterinary Diagnostic Laboratory (ISU VDL) from 2018 to 2024. Antemortem and postmortem sample types were selected and analyzed based on reverse transcription real-time polymerase chain reaction (RT-rtPCR) cycle threshold (Ct) values. The Ct values corresponding to 95%, 75%, and 50% probabilities of sequencing or virus isolation success were determined for each sample type. For antemortem samples, a 95% probability of success for HA Sanger sequencing on nasal swabs exhibited a Ct value of 27.8 from 1046 samples and 23.6 for NA sequencing based on 66 nasal swabs. Using oral fluids, HA and NA Sanger sequencing success was at Ct values of 27.3 from 3446 samples and 22.1 from 137 samples, respectively. For postmortem samples, lung tissue had the highest number of sequences for the HA and NA, with Ct values of 25.7 and 21.5, respectively. For a 95% probability of successful virus isolation, nasal swabs demonstrated a Ct value of 21.1 from 647 samples, while lungs had a Ct value of 18.7 from 5892 samples. This study determined that nasal swabs and lung tissue had the highest probability of IAV gene sequencing and virus isolation success, while oral fluids, a common swine diagnostic sample type that is easy to collect and welfare-friendly, can be effective for gene sequencing when using lower IAV RT-rtPCR Ct values, i.e., ≤27.3. These results provide practical expectations for successful IAV HA and NA gene sequencing and virus isolation at 95%, 75%, and 50% probabilities based on sample type and RT-rtPCR Ct values to improve diagnostic testing strategies in swine populations.

## Simple Summary

Influenza A virus is frequently detected in pigs worldwide, and surveillance through diagnostic testing is important for understanding virus circulation on farms. This study assessed different sample types and the probability of successfully identifying and growing the virus in the veterinary diagnostic laboratory. Scientists analyzed thousands of samples submitted to a diagnostic laboratory between 2018 and 2024. Nasal swabs and lung tissue samples had the best results, with a high chance of successfully identifying and isolating the virus when present in sufficient quantities. Oral fluids, which are easier and less stressful to collect from pigs, also worked well, but virus levels had to be much higher. These findings help veterinarians and researchers choose the best sample types for influenza A virus surveillance and understand how likely they are to obtain useful results from testing based on the amount of virus present in the sample. This can better inform veterinarians before requesting diagnostic tests, such as sequencing and virus isolation, leading to more effective monitoring in pigs, which is important for animal health and for preventing possible spread to humans.

## 1. Introduction

Influenza A virus (IAV) is a member of the *Orthomyxoviridae* family and has a genome consisting of linear, negative-sense, single-stranded RNA divided into eight segments [1]. The virus is enveloped, and its surface is characterized by glycoproteins that include hemagglutinin (HA) and neuraminidase (NA). The envelope surrounds eight helically symmetrical nucleocapsid gene segments of different sizes, including PB2 (Polymerase Basic 2), PB1 (Polymerase Basic 1), PA (Polymerase Acidic), HA (Hemagglutinin), NP (Nucleoprotein), NA (Neuraminidase), M (Matrix), and NS (Non-Structural protein) [2,3].

IAV is a significant pathogen in swine production systems, posing threats to animal health, production efficiency, and public health due to its zoonotic potential [4]. For instance, the 2009 H1N1 influenza pandemic, an IAV lineage currently endemic in humans and swine, resulted in substantial economic losses to the US pork industry, estimated at over USD 1 billion, and public misperceptions about pork safety [5]. Recent cases of Highly Pathogenic Avian Influenza (HPAI) H5N1 in dairy cattle and cats, as well as the detection of other spillover events in a broad host range, are concerning and suggest an increasing potential for other subtypes of the virus to adapt to mammals, including livestock, pets, and humans [6,7].

The recent H5N1 cases that have crossed species barriers highlight the need for robust surveillance and improved diagnostic strategies to monitor current endemic strains of IAV as well as emerging subtypes of the virus in the swine industry, ensuring early detection to inform control measures. A recent study showed that pigs are susceptible to H5N1 infection and highlighted the importance of biosecurity in swine herds to protect against virus incursions [8]. Furthermore, the recent H5N1 detection in swine from a backyard farm in the United States highlighted the increasing risk of the virus to the swine industry [9]. Therefore, accurate and timely IAV gene sequencing is critical to improve diagnostic surveillance for the rapid and successful detection of endemic strains of IAV or the detection of potential H5N1 incursions that may occur in commercial swine farms.

Sequencing IAV gene segments allows for characterizing current strains or newly emerging viruses, as demonstrated with the 2009 H1N1 pandemic outbreak [10]. However, the success of gene sequencing by Sanger methods depends highly on the sample type and other associated factors, including the RT-rtPCR Ct value that represents a semi-quantitative amount of target in the sample as well as sample collection methods, and sample handling, which, taken together, can influence sequencing outcomes [11].

Despite advancements in sequencing technologies, a research gap remains in understanding how factors associated with common antemortem and postmortem sample types, such as oral fluids, nasal swabs, and lung tissues, may impact IAV sequencing success. These may include variations in the quality of the sample type and Ct value at the time of testing by RT-rtPCR. Comparing the variability in viral load across sample types based on Ct value and linking this finding to IAV sequencing success helps establish realistic expectations for additional diagnostic testing and potential justification of the added expense.

Virus isolation remains an important tool for obtaining IAV isolates for further characterization, including antigenic analysis and pathogenicity studies, or most importantly, for vaccine production [12]. Unlike PCR-based detection, which identifies viral RNA regardless of virus viability, virus isolation allows the recovery of live IAV [13]. However, the success of virus isolation is highly dependent on the sample type [14], viral load in the sample at the time of collection [15], and sample handling conditions [16].

Given these factors, understanding the relationship between sample type, viral load, and IAV sequencing or isolation success is critical for the selection of additional diagnostic tests. Therefore, the objective of this study was to evaluate the probability of IAV HA and NA Sanger sequencing and virus isolation success across different sample types based on RT-rtPCR Ct values from 2018 to 2024. Diagnostic samples were passively submitted and tested at the ISU VDL, considering selected antemortem and postmortem samples.

## 2. Materials and Methods

### 2.1. Overview of Study Design

This retrospective study targeted samples previously tested for IAV by RT-rtPCR and followed by Sanger sequencing of the HA and NA genes or virus isolation at the ISU VDL. Using RT-rtPCR positive Ct values representing different sample types, such as lung tissues, oral fluids, and nasal swabs, were assessed to determine the 95%, 75% and 50% probability of success at a corresponding Ct value and sample type for optimal sequencing and virus isolation outcomes.

### 2.2. Eligibility Criteria

The eligibility criteria required for each sample type had all diagnostic testing performed at the ISU VDL, including IAV RT-rtPCR, Sanger sequencing, and/or virus isolation. Furthermore, a positive result for IAV RNA by RT-rtPCR was a sample with Ct values lower than 38.

### 2.3. Research Outcome

The primary outcome of this study was to determine the probability of successfully obtaining the IAV HA or NA genes through Sanger sequencing and successful virus isolation using methods validated at the ISU VDL. The probability was assessed based on Ct values that were obtained using a commercial IAV screening RT-rtPCR assay.

### 2.4. Veterinary Diagnostic Laboratory Data

This retrospective study assessed veterinary diagnostic laboratory (VDL) data on porcine samples submitted to the ISU VDL between 2018 and 2024 for IAV screening using RT-rtPCR. Samples that tested positive (Ct value < 38) for IAV by RT-rtPCR were analyzed for HA (H1 or H3) and NA (N1 or N2) Sanger sequencing and virus isolation. The dataset used for is study only included H1 and H3 HA Sanger sequencing results, as no H5N1 IAV were detected in commercial swine farms during the study period from 2018 to 2024. The samples were also classified as antemortem or postmortem. The antemortem samples were nasal swabs, udder wipes, nasal wipes, and oral fluids. The postmortem samples were lung tissues, bronchial swabs, bronchoalveolar lavage, and tracheal swabs.

Data were retrieved from the ISU VDL database, including submission metadata (e.g., sample type, submission date), Sanger sequencing results (HA or NA successful or unsuccessful and corresponding Ct values), and virus isolation results (successful or unsuccessful and corresponding Ct values). Sanger sequencing results (HA or NA) were considered successful only when HA (1695–1701 bp) or NA (1410 bp) gene sequences of full length without ambiguous bases were obtained following the standard procedures of the ISU VDL. Sanger sequencing of the HA and/or NA genes was performed according to standard operating procedures of the ISU VDL [17]. Additionally, virus isolation was considered successful when the virus isolate was confirmed by observing cytopathic effect (CPE) in MDCK cells consistent with influenza virus replication and detection of hemagglutination units, following the standard procedures of the ISU VDL [18].

### 2.5. Statistical Analyses

All statistical analyses were conducted in R statistical software [19]. Three binary outcomes were analyzed separately: (i) the successful Sanger-sequencing of the haemagglutinin (HA) gene, (ii) the successful sequencing of the neuraminidase (NA) gene, and (iii) the successful isolation of live virus. For each outcome, a binomial response variable (successful or unsuccessful) was regressed on the RT-rtPCR cycle-threshold (Ct, continuous) and sample type (categorical) using a generalized linear model (GLM) with binomial errors and a logit link. Estimated marginal means and pair-wise contrasts among sample types were obtained with the *emmeans* package [20]. All graphics were produced with the ggplot2 package [21].

To identify the Ct value associated with a prespecified probability of success (X = 95%, 75% or 50%), a dense grid of 1000 equally spaced Ct values spanning the empirical range was built for every sample type using the dplyr package [22]. For each grid point, the fitted linear predictor and corresponding standard error were extracted using the predict () function. These were back-transformed with the inverse-logit to yield the fitted probability and its Wald 95% confidence limits. The grid row (with Ct values) whose predicted probability was nearest X% defined the point estimate, whereas the rows where the lower and upper Wald bands intersected X% became the lower and upper confidence limits.

Model discrimination was quantified with the area under the receiver-operating-characteristic curve (AUC), calibration was assessed with the Hosmer–Lemeshow goodness-of-fit χ^2^ test (10 risk deciles), while the overall explained variance was expressed as Nagelkerke’s pseudo-R^2^. These were computed with the pROC [23], ResourceSelection [24], and DescTools [25] packages.

## 3. Results

### 3.1. HA Sanger Sequencing Results

**Figure 1:**
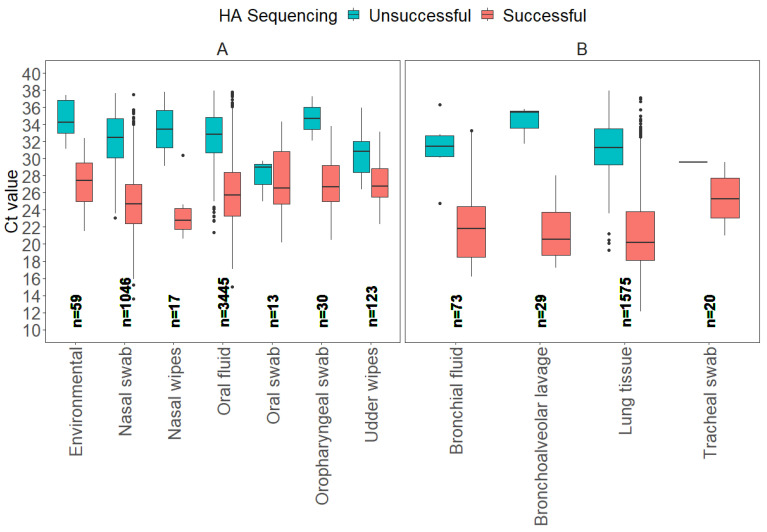
Boxplots display the distribution of influenza A virus RT-rtPCR Ct values for each sample type that were determined successful or unsuccessful for HA Sanger sequencing at the ISU VDL, including antemortem samples (**A**) and postmortem samples (**B**).

From 2018 to 2024, a total of 7871 HA sequences were generated at the ISU VDL, of which 1137 samples were considered not eligible, as they were not tested by RT-rtPCR at the ISU VDL. As a result, 6734 samples had the entire testing process completed at the ISU VDL, from IAV RNA detection by RT-rtPCR to HA sequencing. Next, from the 6734 eligible samples, 304 samples were categorized as “other”, including virus isolates and “fluid” samples of unspecified origin, resulting in 6430 known clinical sample types that were analyzed. The box plots illustrate the distribution of Ct values for all sample types divided into antemortem and postmortem samples (Figure 1).

From a total of 6430 samples, seven antemortem sample types (environmental, nasal swab, nasal wipes, oral fluid, oral swab, oropharyngeal swab, udder wipes) accounted for 73.6% (4733) of the samples tested for HA Sanger sequencing, and four postmortem (bronchial fluid, bronchoalveolar lavage, lung tissue, tracheal swab) samples accounted for 26.4% (1697). From a total of 5737 successful HA sequences, oral fluid represented 52.0% (2982), lung 25.8% (1483), nasal swab 16.8% (1046), udder wipes 1.7% (123), bronchial fluid 1.2% (67), environmental 0.8% (48), oropharyngeal swab 0.5% (28), bronchoalveolar lavage 0.5% (26), tracheal swab 0.3% (19), nasal wipes 0.2% (11), and oral swab 0.2% (10).

For the model evaluation for HA gene Sanger sequencing, the binomial GLM showed strong discrimination (AUC = 0.91 [95% CI: 0.90, 0.92]), adequate calibration (Hosmer–Lemeshow χ^2^ = 5.0, *p* = 0.76), and moderate explanatory power (Nagelkerke R^2^ ≈ 0.48). The ROC curve detection model on HA Sanger sequencing is provided in Supplemental material (Supplemental Figure S1). The Ct values for successful HA Sanger-sequenced antemortem samples, such as nasal swabs, oral fluids, and oropharyngeal swabs, displayed broad Ct ranges for successful results, with nasal swabs showing the widest range of Ct values between 13.6 and 37.5. Postmortem samples, including bronchial fluid, bronchoalveolar lavage, and lung tissue, had average Ct values for HA Sanger sequencing success that were generally lower compared to antemortem samples, with lung tissue showing the lowest minimum Ct value of 12.1. In contrast, average Ct values observed for samples with unsuccessful sequencing were 31.1 for bronchial fluid, 34.3 for bronchoalveolar lavage, 30.8 for lung tissue, and 29.6 for tracheal swab. This variation in Ct values highlights that different sample types may require distinct Ct thresholds for optimal HA sequencing success (Table 1).

**Figure 2:**
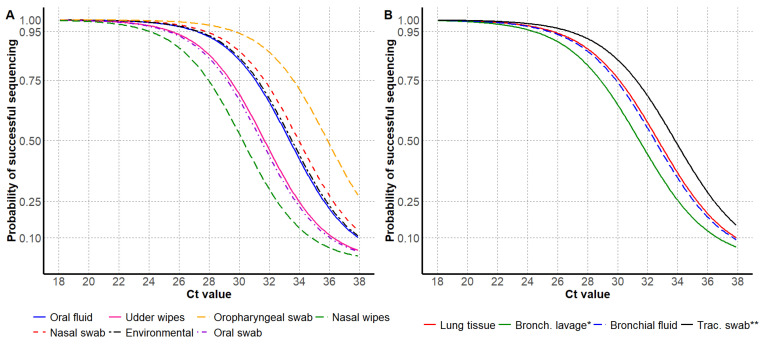
Influenza A virus RT-rtPCR Ct values by the probability of successful HA Sanger sequencing, including antemortem samples (**A**) and postmortem samples (**B**). * Bronchoalveolar lavage; ** Tracheal swab.

For antemortem samples, the 95% probability of HA sequencing success was observed at higher Ct values for oropharyngeal swabs and nasal swabs, and the lowest Ct values were observed for nasal wipes and oral swabs. For postmortem samples, the 95% probability of successful HA sequencing was observed at higher Ct values for tracheal swabs and lung tissue, and the lowest Ct value was observed for bronchoalveolar lavage (Table 2 and Figure 2).

### 3.2. NA Sanger Sequencing Results

**Figure 3:**
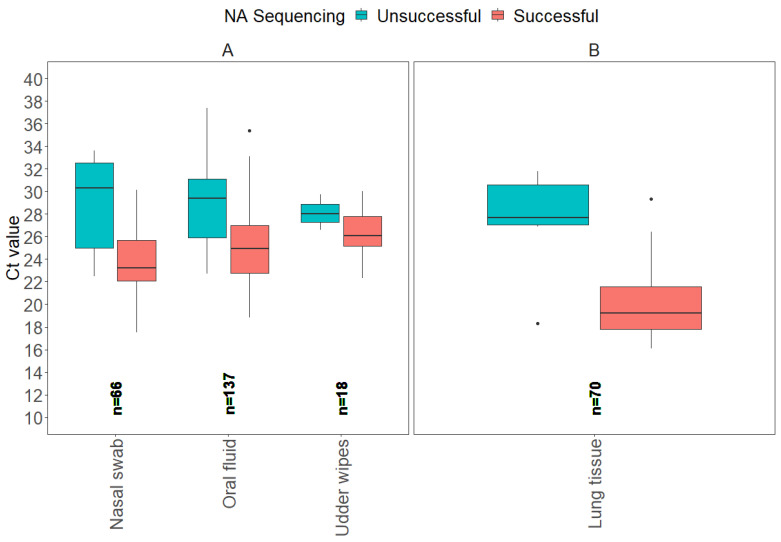
Boxplots display the distribution of influenza A virus RT-rtPCR Ct values for each sample type that were determined successful or unsuccessful for NA Sanger sequencing at the ISU VDL, including antemortem samples (**A**) and postmortem samples (**B**).

From 2018 to 2024, a total of 628 NA gene sequences were analyzed at the ISU VDL, and of those, 322 samples were considered not eligible for the study, as they were not tested by RT-rtPCR at the ISU VDL. From the 307 eligible samples, 15 samples were categorized as “other”, including virus isolates and “fluid” samples of unknown origin. As a result, 291 defined sample types had the entire testing process completed at the ISU VDL, from IAV RNA detection by RT-rtPCR to NA sequencing. The box plots illustrate the distribution of Ct values for all sample types separated by antemortem and postmortem samples (Figure 3).

From a total of 291 samples, 3 antemortem sample types (nasal swab, oral fluid, udder wipes) accounted for 75.9% (221) of the NA Sanger sequences, and lung tissue postmortem samples accounted for 24.1% (70). From a total of 242 samples considered successful for NA sequencing, oral fluid represented 43.0% (104), lung tissue 26.4% (64), nasal swab 24.4% (59), and udder wipes 6.2% (18).

For the model evaluation for NA gene Sanger sequencing, the binomial GLM showed strong discrimination (AUC = 0.84 [95% CI: 0.78, 0.90]), adequate calibration (Hosmer–Lemeshow χ^2^ = 4.5, *p* = 0.81), and moderate explanatory power (Nagelkerke R^2^ ≈ 0.35). The ROC curve detection model for NA Sanger sequencing is provided in Supplemental material (Supplemental Figure S2). The Ct values for NA Sanger sequencing of antemortem samples, such as nasal swabs and oral fluids, showed a broad range of Ct values for successful results, with oral fluid showing the widest range of Ct values between 18.8 and 35.4. Postmortem lung tissue had a lower average Ct value for successful results of 20.1 compared to antemortem sample types, with the lowest minimum Ct value of 16.1. In contrast, the average Ct values among unsuccessful NA sequences had a Ct value of 27.3 for lung tissue. The variability in Ct values suggests the potential of distinct Ct thresholds to influence NA sequencing success based on sample type (Table 3).

**Figure 4:**
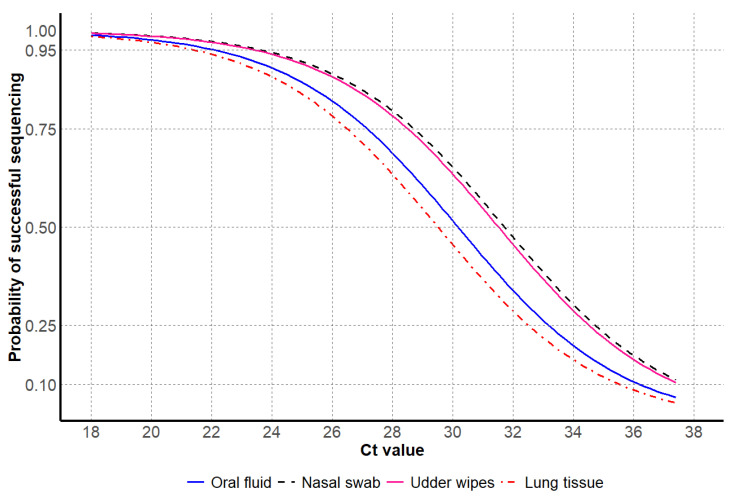
Influenza A virus Ct values by the probability of successful NA Sanger sequencing, including antemortem samples oral fluids, nasal swabs, udder wipes, and postmortem sample lung tissue.

For antemortem samples, the probability of successful NA sequencing was observed at higher Ct values for nasal swabs and udder wipes, in contrast to lower Ct values observed for oral fluids. For postmortem samples, only lung tissues were tested and had lower Ct values at the 95% probability of successful sequencing compared to antemortem samples (Table 4 and Figure 4).

### 3.3. Virus Isolation Results

**Figure 5:**
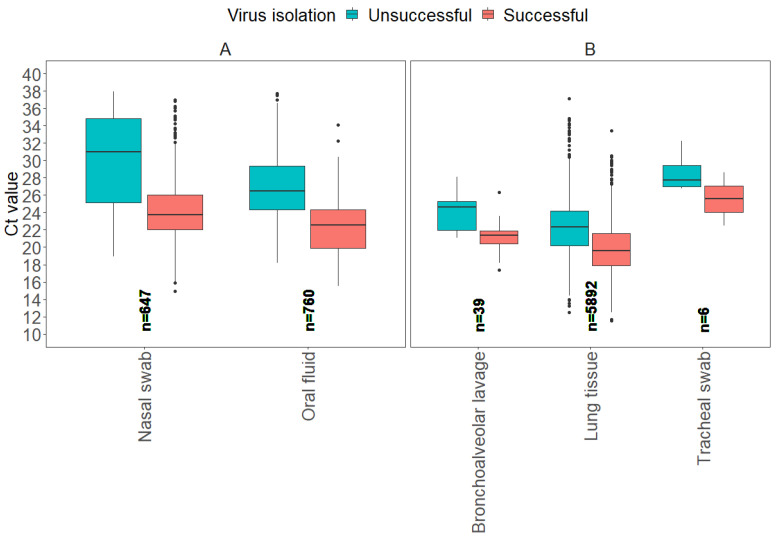
Boxplots display the distribution of influenza A virus RT-rtPCR Ct values for each sample type that were determined successful or unsuccessful for IAV isolation at the ISU VDL, including antemortem samples (**A**) and postmortem samples (**B**).

From 2018 to 2024, a total of 8213 samples were submitted with requests for IAV isolation at the ISU VDL, where 823 samples were considered ineligible for the study, as they were not tested by RT-rtPCR at the ISU VDL. As a result, 7390 samples had the entire testing process completed at the ISU VDL, from IAV RNA detection by RT-rtPCR to virus isolation. Of those, 46 samples were categorized as “other”, including miscellaneous sample types and samples with only one submission. As a result, 7344 samples were evaluated for successful virus isolation. The box plots illustrate the distribution of Ct values for all sample types separated by antemortem and postmortem samples (Figure 5).

From a total of 7344 samples, 2 antemortem sample types (nasal swab, oral fluid) accounted for 19.2% (1407) of those tested for virus isolation, and 3 postmortem sample types (bronchoalveolar lavage, lung tissue, tracheal swab) accounted for 80.8% (5937). From a total of 6147 positive samples for IAV isolation, lung tissue represented 86.6% (5343), nasal swabs 7.8% (483), oral fluid 5.1% (312), bronchoalveolar lavage 0.4% (27), and tracheal swabs 0.03% (2).

For the model evaluation for IAV isolation, the binomial GLM showed strong discrimination (AUC = 0.85 [95% CI: 0.78, 0.91]), adequate calibration (Hosmer–Lemeshow χ^2^ = 5.2, *p* = 0.73), and moderate explanatory power (Nagelkerke R^2^ ≈ 0.37). The ROC curve detection model on virus isolation is provided in Supplemental material (Supplemental Figure S3). The Ct values for IAV isolation from antemortem samples showed a higher average Ct value from nasal swabs compared to oral fluids. Postmortem samples, including bronchoalveolar lavage and lung tissue, had lower average Ct values for successful results compared to antemortem samples, with lung tissue showing the lowest average Ct of 19.7. Regardless of sample type, higher average Ct values, and some with more narrow ranges, were observed if attempts to isolate IAV were unsuccessful. These results suggest that higher Ct values may be associated with reduced virus isolation success (Table 5).

**Figure 6:**
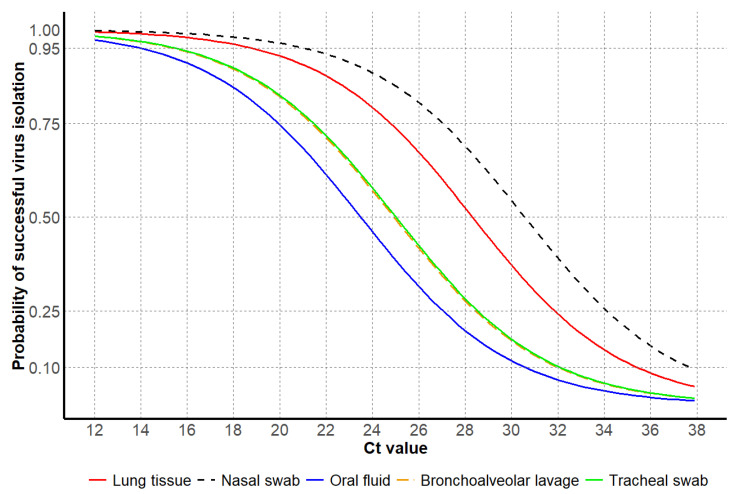
Influenza A virus Ct values based on the probability of successful IAV isolation, including antemortem samples, oral fluids, nasal swabs, and postmortem samples, including lung tissue, bronchoalveolar lavage, and tracheal swab samples.

For antemortem samples, the 95% probability of successful virus isolation was observed at higher Ct values for nasal swabs compared to other antemortem sample types, in contrast to lower Ct values observed for oral fluids. For postmortem samples, lung tissues had higher Ct values at 95% probability of successful virus isolation compared to other postmortem sample types, while bronchoalveolar lavage and tracheal swabs had similar Ct values for the same probability of isolation success (Table 6 and Figure 6).

## 4. Discussion

The findings reported from this observational study provide applicable insights into the probability of successful IAV HA and NA sequencing and virus isolation for different clinical sample types routinely submitted to veterinary diagnostic laboratories. Although the samples were passively submitted, the ISU VDL, a leader in the National Animal Health Laboratory Network (NAHLN), processes over 127,000 cases annually [26], reflecting routine field practices in swine production systems where clinical disease drives diagnostic sampling and testing and where surveillance for important pathogens, including IAV, are also becoming common submissions to veterinary diagnostic laboratories. Furthermore, these data highlight key factors that may influence the success rates of additional diagnostic testing, which include Ct values from the initial RT-rtPCR screening of samples, and should be considered prior to deciding if further testing would be cost-effective. These results also emphasize the importance of sample type selection for diagnostic testing, particularly when targeting HA and NA sequencing or if virus isolation may be necessary, given the potential variability in Ct values across different specimens.

For HA gene Sanger sequencing, antemortem samples showed higher Ct values at 95% probability of successful HA sequencing compared to postmortem samples. This outcome may have been influenced by sample type, considering postmortem samples are typically collected from clinically affected pigs and represent specimens with higher amounts of virus (lower Ct values) that are not used for routine surveillance [27]. The most frequent antemortem sample type for IAV surveillance in swine, among other pathogens submitted for RT-rtPCR testing, has been oral fluids [28]. The results of this study showed that antemortem samples, oral fluids and nasal swabs, had high Ct values for HA Sanger sequencing compared to the postmortem lung tissues at the same probability. Moreover, oral fluids and nasal swabs had a greater number of samples included in the dataset compared to oropharyngeal swabs, thus providing greater confidence in the calculated success rates reported in this study.

Surprisingly, oral fluids had relatively similar Ct values compared to nasal swabs that were consistent with successful HA gene sequencing. This was somewhat unexpected considering PCR inhibitors are often present in oral fluids [29] due to environmental contaminants compared to nasal swabs. However, it is important to consider the difference in the number of oral fluids analyzed in this study, as there was a significantly higher number of oral fluids tested compared to nasal swabs, which may explain differences in the confidence of probability thresholds for these sample types [30]. A recent study [31] showed a higher difference in the proportion of submissions testing for IAV RNA by RT-rtPCR for oral fluids (60%) compared to nasal swabs (6%) and lung tissues (24%), supporting the increase in surveillance for this virus in the US. In addition, oral fluids generally have higher Ct values compared to other sample types as they represent populations [32] with both positive and negative animals contributing to the specimen. A prior study [33] assessed IAV whole genome sequencing using oral fluid samples and observed that successful whole genomes were obtained when the Ct values were lower than 27, which is similar to the results reported in this study that observed a 95% probability of HA sequencing success with a Ct value of 27.3 or lower. Conversely, oral swabs, nasal wipes, and udder wipes showed lower probabilities of sequencing success compared to other antemortem sample types, which may be due to decreased viral loads and potential challenges that may be intrinsic to these sample types. Moreover, these three sample types may be under-represented as they had fewer numbers of sequences included in the analysis, which may have influenced the results and suggests some caution when interpreting the outcomes of this study for samples with fewer numbers [30].

For HA sequencing of postmortem samples, tracheal swabs and lung tissue had lower Ct values at the 95% probability threshold for success compared to the most common antemortem sample types, which include nasal swabs and oral fluids. However, lung tissue was the most frequently tested postmortem sample, accounting for 24.5% of total samples, while tracheal swabs consisted of only 0.3%, suggesting lung tissue may be a more reliable and widely used postmortem sample for HA sequencing. Indeed, lung tissues are more likely to be submitted from pigs clinically affected with IAV, with a potential of low RT-rtPCR Ct values that perhaps increases the overall success observed for this sample type, presenting a potential bias on the results of this observational study.

There was a significantly lower number of submissions requesting NA gene sequencing, likely due to the predominant focus on using HA sequences for epidemiological investigations and evaluating nucleotide homologies between different strains of IAV. However, there has been a recent increase in the number of NA sequencing requests compared to previous years as a result of its potential use in vaccines due to its ability to induce protection against infection and clinical disease [34]. For NA sequencing, oral fluids exhibited the highest number of tested samples, followed by nasal swabs. The nasal swabs demonstrated the highest Ct value in contrast to oral fluids, which demonstrated the lowest Ct value required for a 95% probability of successful NA sequencing. These results indicate that nasal swabs may be the ideal antemortem sample type for successful NA sequencing, with oral fluids requiring a slightly lower Ct value, or higher virus concentration, for sequencing success. Udder wipes, while representing a smaller proportion of tested samples, had a comparable Ct value to the nasal swabs, indicating that they may also serve as a reliable but less commonly used sample type for IAV sequencing and a potential option for breeding herd surveillance [35]. For postmortem samples, lung tissue consisted of 24.1% of total samples for NA sequencing and had a lower Ct value at 95% sequencing success compared to HA sequencing, also emphasizing its reliability for NA sequencing. These findings highlight nasal swabs and lung tissue as important sample types for NA sequencing success, with oral fluids also offering a potential alternative for antemortem sampling when the Ct value is approximately 22.1 or lower, to achieve a 95% success rate.

Samples with RT-rtPCR Ct values above 30, based on the test used at the ISU VDL, demonstrated a decreased probability of successful sequencing and virus isolation, indicating a higher risk of failure at lower target concentrations. In addition, since Ct values from RT-rtPCR help interpret the significance of the result, veterinarians often request sequencing or virus isolation on samples with lower Ct values to increase the likelihood of diagnostic success [36]. These results suggest that as Ct values increase, reflecting lower concentrations of the pathogen, the likelihood of obtaining usable sequencing data or isolating the virus diminishes. The relationship also varied by sample, antemortem or postmortem, and sample type, highlighting the importance of sample matrix and pathogen concentration in downstream success when additional diagnostic testing is necessary.

Virus isolation typically requires lower Ct values for success compared to gene sequencing. For virus isolation, lung tissues were the most common sample type, representing 80% of all samples tested, and represented the postmortem sample with a higher Ct value at 95% probability of successful virus isolation. For lung tissue samples submitted for virus isolation, 9% of the samples were unsuccessful, with an average Ct value of 22.5 and the lowest Ct value of 12.5. While Ct value is an important criterion for successful sequencing and virus isolation, it is important to consider that even an ideal sample type, such as lung tissue, could be unsuccessful due to multiple factors. For example, besides Ct values and sample types, other potential factors that may influence the success of virus isolation could be stage of the disease process and age of the animals sampled [11], clinical signs present at the sampling time [37], and sample selection, collection methods, and submission conditions prior to receipt and testing at the VDL [27]. Furthermore, identifying the cause of disease may require multiple or repeated submissions and/or tests, and interpreting results in the context of the farm’s history, clinical signs of the population, and pathology present is essential [38]. Therefore, negative virus isolation results, regardless of sample type, should be interpreted with caution as it does not confirm the absence of the virus, only that it was not successfully isolated.

For antemortem samples, nasal swabs had higher virus isolation success with a higher Ct value at 95% probability compared to other antemortem sample types. In contrast, oral fluids required the lowest Ct value, indicating success of this sample type requires high viral loads in the sample, as suggested by the average Ct value of 13.9 at 95% probability of success. This finding agrees with a previous study, which observed that virus isolation was much less successful from oral fluids compared to nasal swabs, which may be associated with a large amount of anti-IAV antibodies in oral fluid, [39] and glycoproteins which have inhibiting and neutralizing activities against the virus, or due to the poor quality of the sample type with environmental components that often disrupt cell culture [40]. Postmortem samples, particularly lung tissue, presented the highest probability of yielding a virus isolate, with a successful Ct value of 18.7 or lower. This finding suggests that nasal swabs and lung tissue are more reliable sample types for surveillance programs or diagnostics where the primary goal is to obtain a virus isolate, perhaps for vaccine use, antibody assays, or to obtain a whole genome sequence.

The findings from this study can also inform IAV sequencing strategies in the event of emerging influenza strains in the swine industry, as a hypothetical H5N1 emergence. By understanding the effectiveness of different antemortem and postmortem sample types for successful IAV sequencing, these results can guide veterinarians and producers in optimizing surveillance protocols and diagnostic approaches when rapid characterization of novel strains is necessary, including the emergence of IAV in swine that spill over from other species [41]. This is particularly relevant for early detection and rapid response efforts, where selecting appropriate sample types and testing methods can improve surveillance sensitivity and outbreak management [42]. In addition, the methodology used in this study can support broader applications across other pathogens, sample types, and diagnostic contexts in swine. Finally, the methodology used in this study can be extrapolated to other veterinary diagnostic laboratories, but with some caution due to differences in sample processing, RNA extraction methods, differences in RT-rtPCR, Sanger sequencing, and virus isolation techniques.

This retrospective study limited the ability to evaluate specific sample pre-treatment strategies that may improve sequencing success. Enrichment methods such as sample dilution, centrifugation, or host RNA depletion have been shown to improve viral RNA concentration and sequencing [43,44]. While these approaches were not assessed in this study, their potential use is worth considering, particularly for sample types associated with higher RT-rtPCR Ct values and lower sequencing success. Thus, future prospective studies should explore the impact of such pre-analytical treatments on sequencing efficiency to improve the reliability of sequencing for diagnostic and surveillance purposes.

This study is subject to some limitations. First, samples are passively submitted to the diagnostic laboratory for multiple purposes without knowledge of sample collection, transportation, storage conditions, or handling processes. As passive submissions to a veterinary diagnostic laboratory, the data used in this study includes a blend of samples from clinically affected pigs and submissions for routine surveillance that do not necessarily represent all diagnostic scenarios, potentially introducing selection bias [45] that may not fully reflect the general swine population. In addition, veterinarians and diagnosticians typically select samples with lower RT-rtPCR Ct values once testing is completed to bias sequencing and virus isolation success. Therefore, sample types with higher Ct values were represented by lower numbers of samples, which may have influenced the results of this analysis. In addition, these results do not provide context regarding differences that may exist between swine ages that may, in turn, also influence the success of IAV detection, sequencing, and virus isolation. Additionally, when utilizing VDL data, it is essential to recognize that the test results are based on samples submitted specifically for diagnostic purposes and, thus, do not represent IAV prevalence or incidence in the population.

## 5. Conclusions

This study identified nasal swabs and lung tissue as the samples with the highest probability of successful IAV sequencing by Sanger techniques and virus isolation, while oral fluids, a common swine diagnostic sample type that is easy to collect and welfare-friendly, can be effective for sequencing when lower Ct values, i.e., 27.3 or lower, are detected in the sample. These findings provide critical insights into different sample types and RT-rtPCR Ct value thresholds at 95%, 75%, and 50% probabilities of success for porcine antemortem and postmortem samples, informing clinical and surveillance strategies aimed at improving the success of influenza diagnostic testing in swine after a positive IAV RNA tested by RT-rtPCR.

## Figures and Tables

**Table 1:** Influenza A virus RT-rtPCR average Ct values and distribution for successful and unsuccessful HA gene Sanger sequencing.

Sample Type	HA Sequencing Successful	HA Sequencing Unsuccessful
**Antemortem Samples**	**Ct Value: Average (Min–Max)**	**Ct Value: Average (Min–Max)**
Environmental	27.2 (21.5–32.4)	34.6 (31.1–37.4)
Nasal swab	24.9 (13.6–37.5)	32.1 (23.1–37.6)
Nasal wipes	23.7 (20.6–30.4)	33.4 (29.1–37.8)
Oral fluid	25.9 (15.0–37.8)	32.4 (21.4–37.9)
Oral swab	27.3 (20.2–34.3)	27.9 (25.0–29.7)
Oropharyngeal swab	27.2 (20.5–33.8)	34.7 (32.1–37.3)
Udder wipes	27.2 (22.3–33.1)	30.6 (26.4–35.9)
Postmortem samples		
Bronchial fluid	22.1 (16.2–33.3)	31.1 (24.8–36.3)
Bronchoalveolar lavage	21.5 (17.2–28.0)	34.3 (31.7–35.8)
Lung tissue	21.3 (12.1–37.1)	30.8 (19.3–37.9)
Tracheal swab	25.4 (21.0–29.6)	29.6 (25.0–34.6)

**Table 2:** Influenza A virus RT-rtPCR Ct values based on different probabilities of HA gene Sanger sequencing success by sample type.

Sample Type	Ct Value by Probability of Success (C.I. ^1^)			Total Number	Proportion
**Antemortem Samples**	**95%**	**75%**	**50%**		
Environmental	27.4 (25.4, 29.0)	31.2 (29.4, 32.9)	33.5 (31.8, 35.3)	59	0.9%
Nasal swab	27.8 (27.1, 28.4)	31.7 (31.1, 32.4)	34.0 (33.4, 34.8)	1046	16.3%
Nasal wipes	24.1 (18.8, 29.2)	28.0 (22.7, 33.1)	30.3 (25.1, 35.5)	17	0.3%
Oral fluid	27.3 (26.7, 27.5)	31.1 (30.8, 31.4)	33.3 (33.1, 33.8)	3445	53.6%
Oral swab	25.4 (21.7, 28.7)	29.0 (25.7, 32.7)	31.5 (28.1, 34.9)	13	0.2%
Oropharyngeal swab	29.8 (26.1, 33.3)	33.6 (30.1, 37.2)	35.9 (32.4, 37.9)	30	0.5%
Udder wipes	25.5 (24.3, 26.6)	29.4 (28.3, 30.5)	31.7 (30.6, 32.9)	123	1.9%
Postmortem samples					
Bronchial fluid	25.6 (23.6, 28.3)	29.9 (27.5, 32.3)	32.5 (29.8, 34.6)	73	1.1%
Bronchoalveolar lavage	24.9 (20.8, 28.9)	28.8 (24.7, 32.9)	31.4 (27.1, 35.2)	29	0.5%
Lung tissue	25.7 (25.4, 26.7)	30.1 (29.4, 30.6)	32.7 (31.7, 33.0)	1575	24.5%
Tracheal swab	26.9 (22.5, 31.4)	31.3 (26.5, 35.3)	33.8 (28.9, 37.7)	20	0.3%
Total				6430	100%

^1^ 95% Confidence Interval.

**Table 3:** Influenza A virus RT-rtPCR average Ct values and distribution for successful and unsuccessful NA gene Sanger sequencing.

Sample Type	NA Sequencing Successful	NA Sequencing Unsuccessful
**Antemortem Samples**	**Ct Value: Average (Min–Max)**	**Ct Value: Average (Min–Max)**
Nasal swab	23.9 (17.5–30.1)	28.8 (22.5–33.6)
Oral fluid	25.1 (18.8–35.4)	29.0 (22.7–37.4)
Udder wipes	26.5 (22.3–30.0)	28.1 (26.6–29.7)
Postmortem samples		
Lung tissue	20.1 (16.1–29.3)	27.3 (18.3–31.8)

**Table 4:** Influenza A virus RT-rtPCR Ct values based on different probabilities of NA gene Sanger sequencing success by sample type.

Sample Type	Ct Value by Probability of Success (C.I. ^1^)			Total Number	Proportion
**Antemortem Samples**	**95%**	**75%**	**50%**		
Nasal swab	23.6 (20.6, 26.1)	28.8 (26.3, 31.4)	31.8 (29.2, 35.1)	66	22.8%
Oral fluid	22.1 (19.3, 23.8)	27.1 (25.8, 28.4)	30.1 (28.9, 32.0)	137	47.2%
Udder wipes	23.4 (19.3, 27.1)	28.6 (25.0, 32.4)	31.6 (28.1, 36.0)	18	5.9%
Postmortem samples					
Lung tissue	21.5 (18.1, 24.1)	26.4 (23.8, 29.5)	29.4 (26.8, 33.1)	70	24.1%
Total				291	100%

^1^ 95% Confidence Interval.

**Table 5:** Influenza A virus RT-rtPCR average Ct values and distribution for successful and unsuccessful virus isolation.

Sample Type	Virus Isolation Successful	Virus Isolation Unsuccessful
**Antemortem Samples**	**Ct Value: Average (Min–Max)**	**Ct Value: Average (Min–Max)**
Nasal swab	24.3 (14.9–37.0)	30.1 (18.9–37.9)
Oral fluid	22.6 (15.5–34.1)	26.9 (18.2–37.7)
Postmortem samples		
Bronchoalveolar lavage	21.1 (17.4–26.3)	24.1 (21.1–28.1)
Lung tissue	19.7 (11.5–33.4)	22.5 (12.5–37.1)
Tracheal swab	25.5 (22.5–28.6)	28.6 (26.8–32.2)

**Table 6:** Influenza A virus RT-rtPCR Ct values based on different probabilities of successful IAV isolation by sample type.

Sample Type	Ct Value by Probability of Success (C.I.^1^)		Total Number		Proportion
**Antemortem Samples**	**95%**	**75%**	**50%**		
Nasal swab	21.1 (20.1, 21.9)	27.0 (26.3, 27.8)	30.6 (29.9, 31.4)	647	8.8%
Oral fluid	13.9 (12.9, 14.9)	20.0 (19.3, 20.5)	23.5 (22.9, 24.0)	760	10.3%
Postmortem samples					
Bronchoalveolar lavage	15.5 (13.0, 17.8)	21.4 (19.0, 23.7)	24.8 (22.6, 27.3)	39	0.5%
Lung tissue	18.7 (18.4, 19.2)	24.8 (24.4, 25.1)	28.3 (27.8, 29.0)	5892	80.2%
Tracheal swab	15.5 (11.5, 21.4)	21.4 (15.5, 27.4)	25.1 (19.1, 31.0)	6	0.1%
Total				7344	100%

^1^ 95% Confidence Interval.

## Data Availability

The datasets produced and analyzed during the current study are included in this research article. Further information can also be obtained by contacting the corresponding author.

## Supplementary Materials

The following supporting information can be downloaded at: https://www.mdpi.com/article/10.3390/vetsci12070683/s1, Figure S1: Receiver Operating Characteristic (ROC) curve detection model for HA Sanger sequencing; Figure S2: Receiver Operating Characteristic (ROC) curve detection model for NA Sanger sequencing; Figure S3: Receiver Operating Characteristic (ROC) curve detection model for virus isolation.
