# Supplementary material for: Porcine Sample Type Characteristics Associated with Sequencing and Isolation of Influenza A Virus
Source: Vet Sci. 2025 Jul 19;12(7):683. doi: 10.3390/vetsci12070683 (PMC12298594; doi:10.3390/vetsci12070683)
Supplement: Supplementary file 1 [file vetsci-12-00683-s001.zip › vetsci-3706355-supplementary.pdf]

veterinary sciences

MDPI

Supplemental material

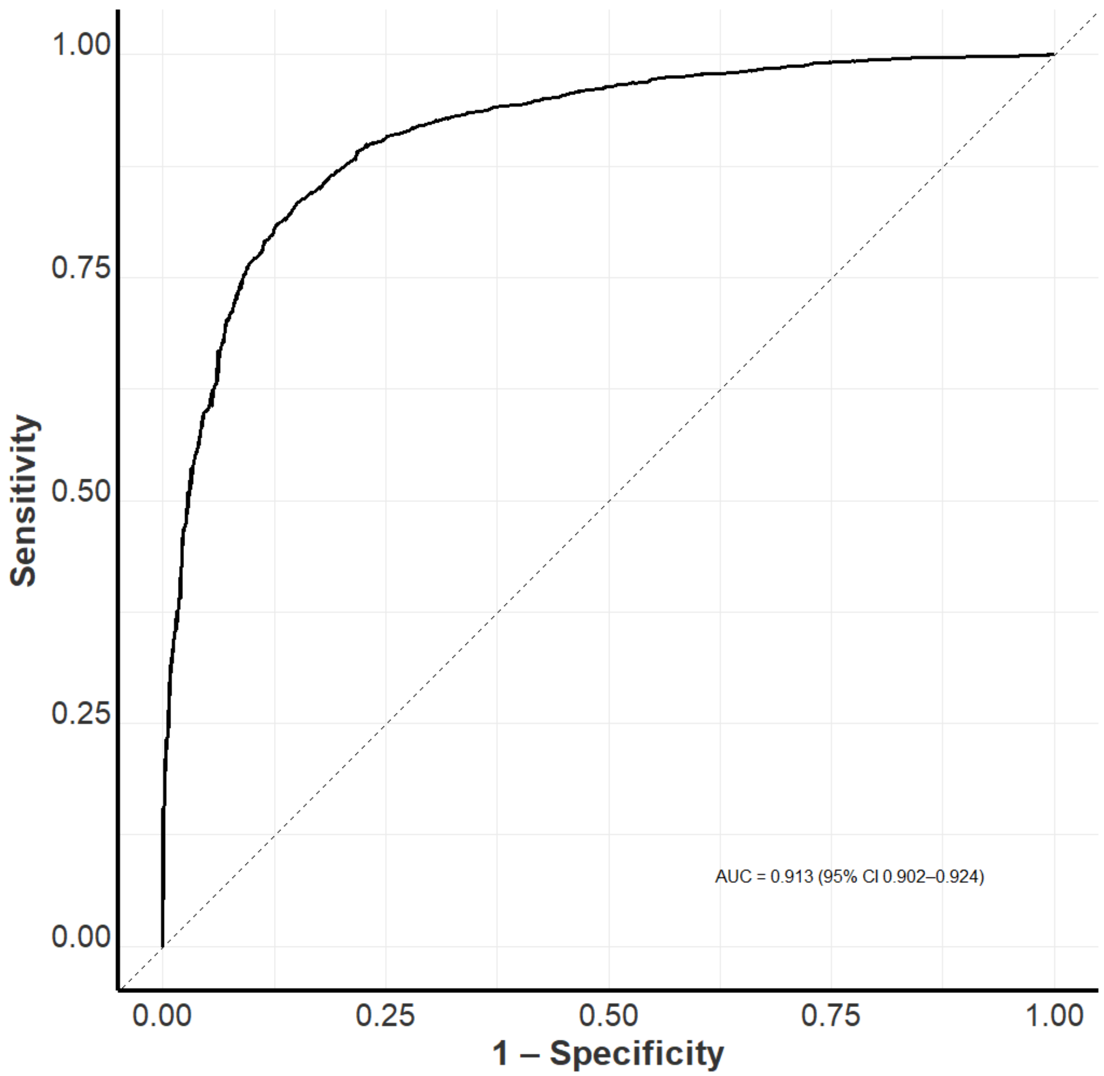

Supplemental Figure S1. Receiver Operating Characteristic (ROC) curve detection model for HA Sanger sequencing.

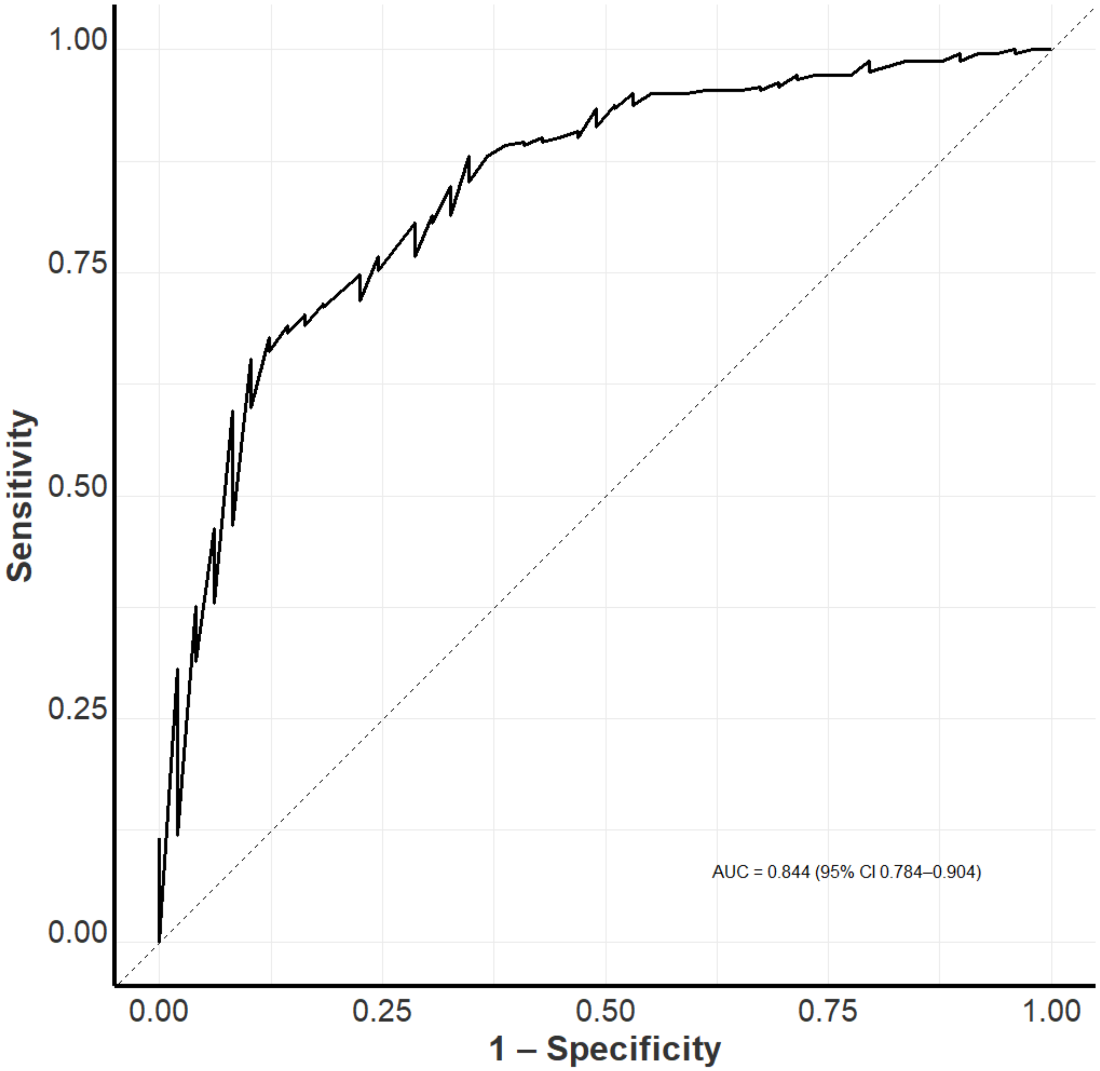

Supplemental Figure S2. Receiver Operating Characteristic (ROC) curve detection model for NA Sanger sequencing.

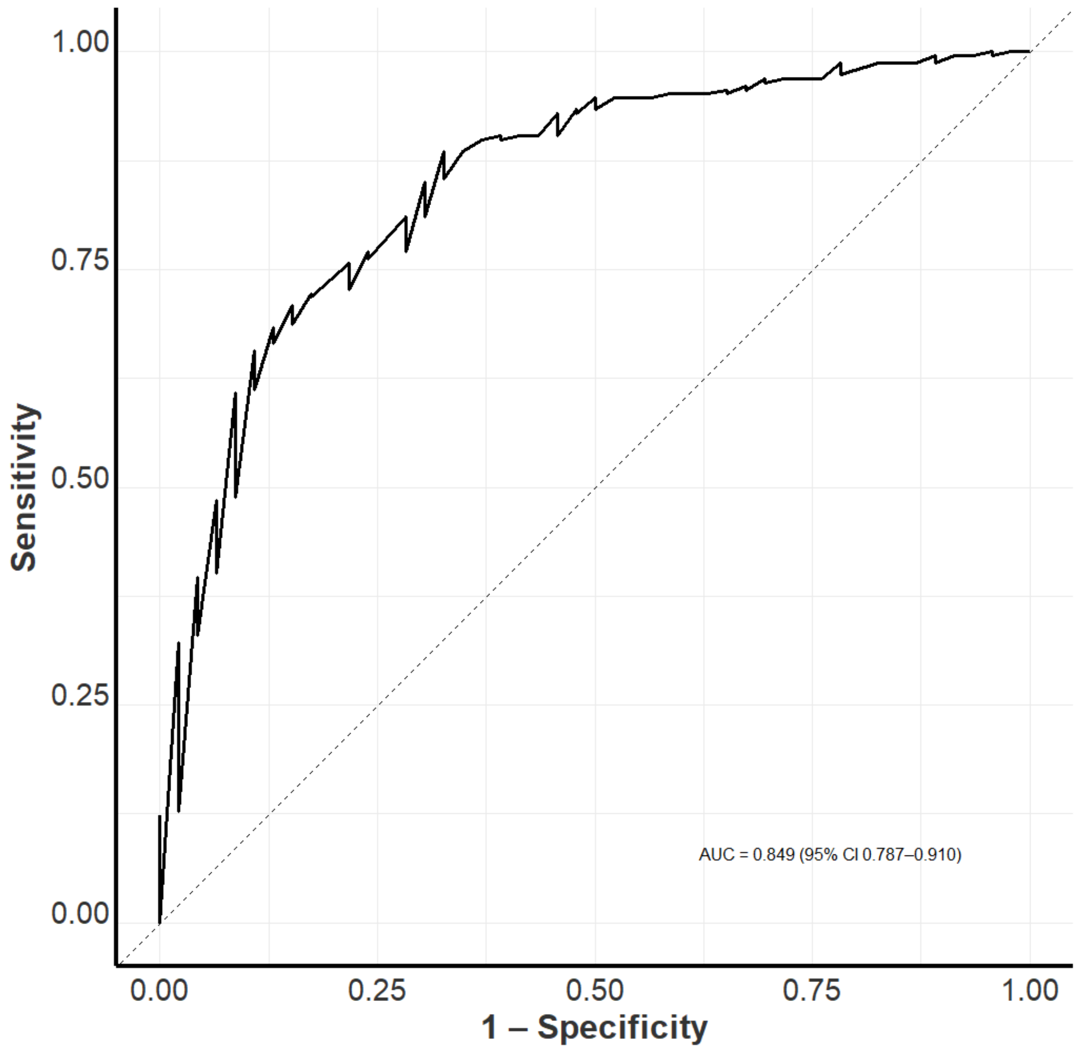

Supplemental Figure S3. Receiver Operating Characteristic (ROC) curve detection model for virus isolation.
